# Supplementary material for: Transalbugineal Artificial Urinary Sphincter: A Refined Implantation Technique to Improve Surgical Outcomes
Source: J Clin Med. 2023 Apr 21;12(8):3021. doi: 10.3390/jcm12083021 (PMC10141998; doi:10.3390/jcm12083021)
Supplement: Supplementary file 1 [file jcm-12-03021-s001.zip › jcm-2277540-supplementary.pdf]

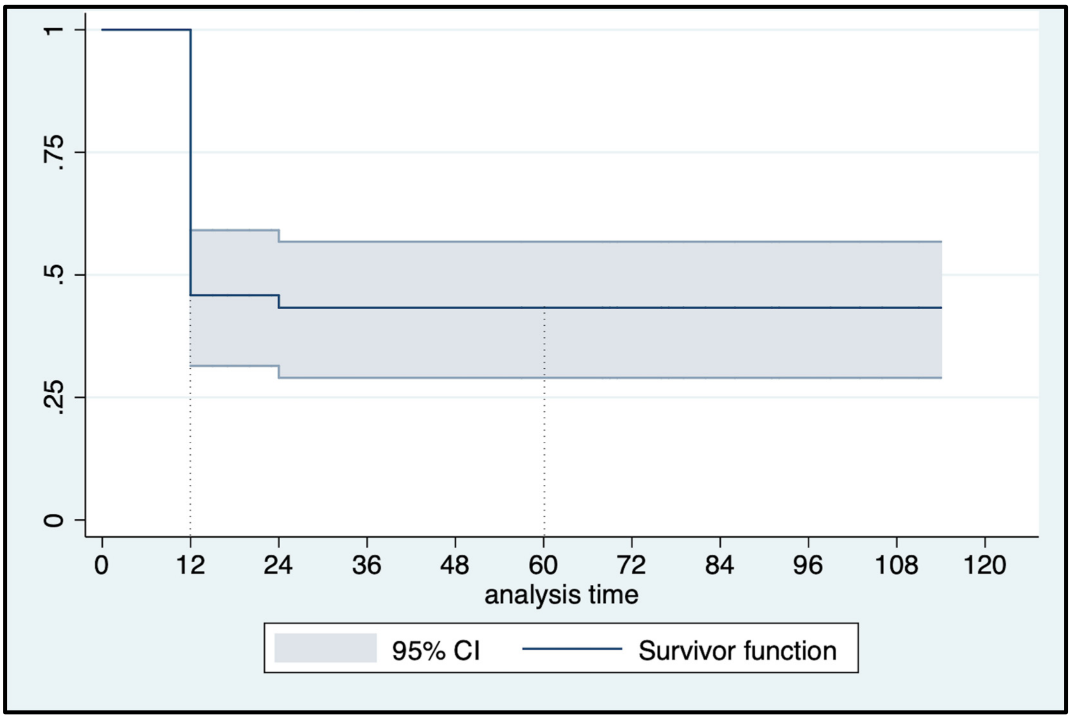

Figure S1. Kaplan-Meier actuarial cure failure-free survival curve.

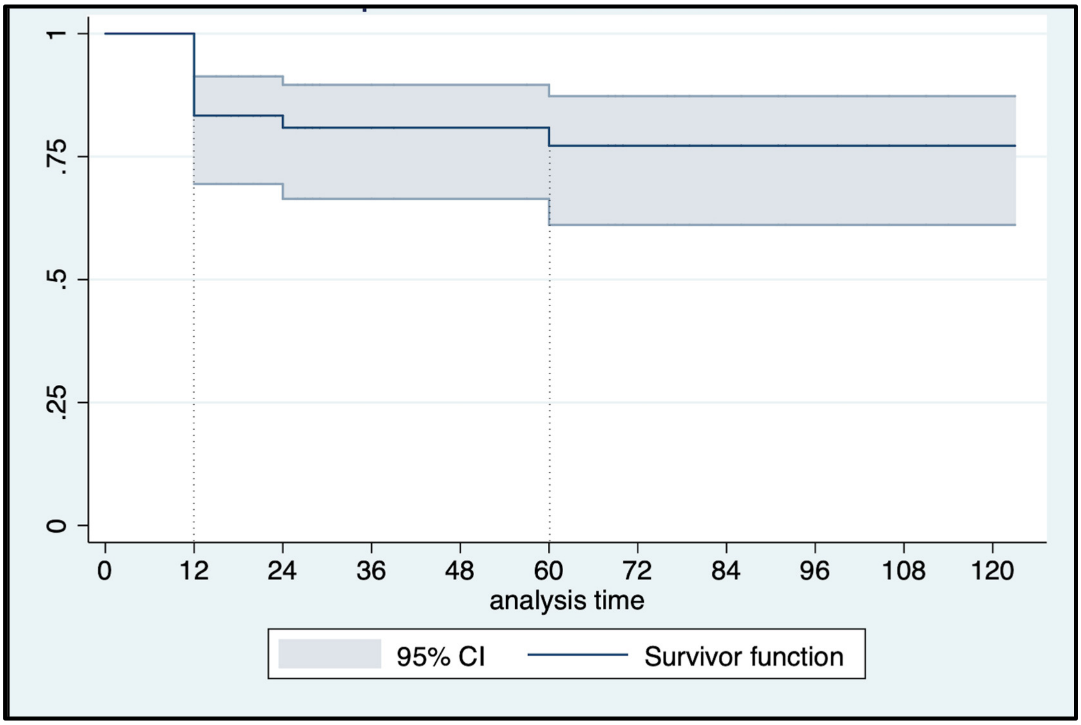

Figure S2. Kaplan-Meier social continence failure-free survival curve.
